# Asymmetric Reduction of Unactivated Alkenes

**DOI:** 10.1002/chem.70826

**Published:** 2026-02-26

**Authors:** Nico D. Fessner, Sebastian Roth, Richard Niese, Michael Müller

**Affiliations:** ^1^ Pharmaceutical and Medicinal Chemistry Institute of Pharmaceutical Sciences University of Freiburg Freiburg Germany

**Keywords:** alkene reduction, biocatalysis, enzymatic reduction, hydrogen atom transfer, unactivated alkenes

## Abstract

The asymmetric reduction of unactivated alkenes remains a key challenge in synthesis due to their inherent lack of polarity and steric bias. Advances range from hydrogenation with molecular hydrogen and precious transition‐metal catalysts, such as Crabtree's Ir and Noyori's Ru systems, to modern approaches employing abundant metals or radical‐mediated hydrogen atom transfer (HAT) under mild conditions. Over the past decades, chemists have assembled a diverse mechanistic toolbox, with each strategy achieving excellent results under specific substrate, selectivity, and operating conditions. Clear trends have emerged toward greater sustainability, lower costs, and operational simplicity. Biocatalysis, previously limited to activated alkenes such as enones, has advanced via engineered promiscuous reductases, photobiocatalysis, and multifunctional enzymes, offering complementary and highly selective transformations. Enzymatic strategies for the reduction of unactivated alkenes, however, are still rare and highly substrate‐specific. Recent innovations, such as BioHAT integrate radical‐based mechanisms into engineered proteins and represent a potential first step toward general and practical biocatalytic solutions. The development of asymmetric reduction of unactivated alkene and synergistic integration of chemical and enzymatic strategies are summarized.

## Graphical abstract

Unactivated alkenes rank among the most inert functional groups in synthesis and their selective reduction remains challenging. This Review charts the evolution from classical metal‐catalyzed hydrogenation to radical hydrogen atom transfer (HAT) and emerging biocatalytic concepts, highlighting how complementary mechanistic strategies, including the recently introduced BioHAT, are reshaping how inert C═C bonds can be reduced.

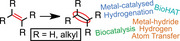

## 1 Introduction

**FIGURE 1:**
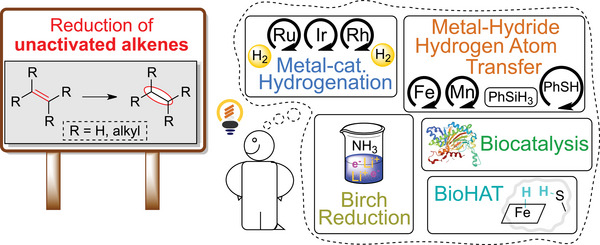
Current strategies for the reduction of unactivated alkenes.

Unactivated C═C bonds, typically electron‐rich and not in conjugation, are intrinsically challenging substrates due to their lack of electronic activation and steric bias [1]. Transition‐metal catalysts, such as Crabtree's iridium complex [2], enable the scalable hydrogenation of unactivated alkenes under mild conditions [3]. Such breakthroughs remain landmarks in synthetic chemistry; however, the scarcity and cost of precious metals call for alternatives [4, 5]. In recent years, the field has progressed from traditional coordinative metal‐catalyzed hydrogenation [6] toward radical‐based methods based on non‐coordinative organometallic chemistry [7, 8]. Innovations in hydrogen atom transfer (HAT) chemistry [9, 10, 11], electrochemistry [12, 13], photochemistry [14, 15], and enzyme catalysis [3, 16] led to mild and more sustainable reduction methods (Figure 1).

Enzymes have gained increasing attention due to their tunability by engineering and compatibility with different reaction conditions, becoming an integral part of today's synthetic toolbox [17, 18]. For example, ene reductases (EREDs) enable the reduction of activated C═C bonds adjacent to electron‐withdrawing groups (EWGs) on a multigram‐scale [19]. The enzymatic reduction of unactivated alkenes remains rare in synthetic application. In nature, such transformations are achieved by strategies including temporary activation (e.g., reversible activation by group installation) or, in some cases, direct alkene reductions [3]. Recently, HAT was imported into an enzyme active site, expanding the biocatalytic repertoire for alkene reduction [20].

This review emphasizes the challenges associated with asymmetric reduction of unactivated alkenes and summarizes existing strategies to overcome them.

## 2 Unactivated Alkene Functionality

**FIGURE 2:**
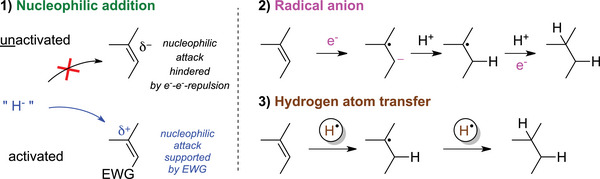
General mechanisms for the reduction of isolated (unactivated) alkenes [1, 24].

Alkene reduction is among the most fundamental reactions in chemistry in controlling chirality in synthesis, as it enables the stereoselective formation of multiple stereocenters. The ease of reduction depends on the degree of activation, influenced by neighboring electronic effects and external factors such as Lewis acids or redox mediators. In general, alkene reduction involves electron donation from a reductant into the molecule's lowest unoccupied molecular orbital. Electron‐deficient (activated) alkenes are readily reduced (Figure 2), while unactivated alkenes lack polarity and are exceptionally inert due to highly negative reduction potentials (typically *E°* < –3 V vs. SCE) [1, 21, 22]. In fact, there are even “*hyperstable*” alkenes known that are completely resistant to hydrogenation [23].

## 3 Chemical Reduction of Unactivated Alkenes

### 3.1 Non‐Catalytic Reduction

**SCHEME 1:**
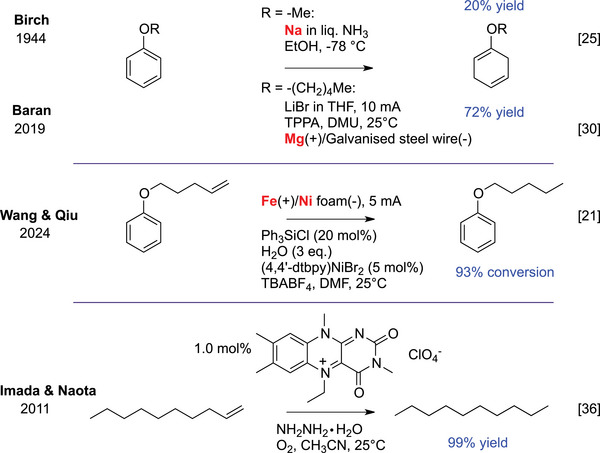
The classical Birch reduction (Birch, 1944), a modern electrochemical variant (Baran, 2019), and an alternative electrochemical protocol that can selectively reduce unactivated alkenes (Wang and Qiu, 2024). The original diimide reduction protocol was advanced with a flavin catalyst by Imada et al.

In 1944, Birch discovered that sodium in liquid ammonia with an alcohol cosolvent could reduce otherwise inert aromatic compounds via solvated electrons (Scheme 1) [25]. Despite its power, the chemofidelity (likelihood of reaction success) and chemoselectivity of dissolving metal reductions are often compromised, as more readily reducible functional groups react preferentially [11]. Safety and sustainability concerns pose further challenges for larger‐scale applications. Recent efforts have produced safer, scalable variants of Birch reduction by replacing ammonia [26, 27], using biocatalytic strategies [28], or driving the reduction with electrical [29, 30], photochemical [15], or mechanochemical [31, 32] energy inputs. Building up on previous work [29, 33], the Baran group developed electrochemically driven Birch‐type reductions that are compatible with a wide range of reductive transformations (Scheme 1) [30]. Chemoselectivity was refined by waveform modulation, which effectively minimized overreduction [34]. In 2024, an electrochemical protocol for selectively reducing unactivated alkenes was reported, using iron cathodes, nickel anodes, and water as the proton source to generate catalytic chlorosilane in situ (Scheme 1) [21]. Although it requires dedicated equipment, the method operates under mild conditions and uses inexpensive reagents, though broader applicability remains to be established.

Another catalyst‐free approach to reducing unactivated alkenes is by diimide (HN═NH) reduction, which proceeds by concerted *syn*‐hydrogenation across the C═C bond. First observed in 1905 but recognized synthetically only decades later [35], the reaction is driven C─H bond formation and the entropically favorable N_2_ release. Diimide is typically generated in situ and provides mild and highly chemoselective alkene reductions. Although diimide is transient and its rapid decomposition can limit scalability, it can selectively reduce alkenes based on substitution, making it useful for substrate‐sensitive systems where metal contamination or over‐reduction of other functional groups is a concern. Recent advances have addressed limitations: aerobic [36] and electrochemical [37] diimide generation allow more controlled reductions. Imada et al. used a synthetic flavin catalyst that is oxidized by O_2_ to generate diimide in situ from hydrazine (Scheme 1) [36], enabling O_2_ to act as a synthetic enabler rather than a destructive oxidant.

### 3.2 Catalytic Hydrogenation

**SCHEME 2:**
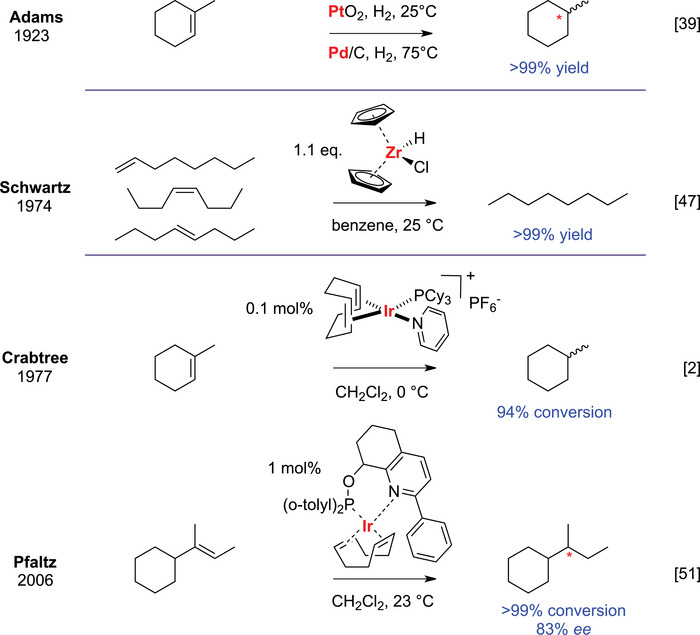
Development of hydrogenation tools for the reduction of unactivated alkenes: heterologous Adams’ catalyst (PtO_2_) and palladium on activated carbon (Pd/C); Schwartz’ reagent used in stoichiometric quantities; Crabtree's achiral catalyst equipped with chirality by Pfaltz.

The hydrogenation of alkenes has undergone a remarkable evolution through more than a century of catalytic innovation. Early industrial Ni‐catalyzed hydrogenation was developed by Boyce, and in the early 1900s, Sabatier demonstrated that finely divided metals such as Ni, Pt, and Pd catalyze the addition of molecular hydrogen to alkenes (1912 Nobel Prize in Chemistry) [38]. This discovery laid the foundation for heterogeneous catalytic hydrogenation, which was further advanced by Adam's catalyst (PtO_2_) developed in 1923 (Scheme 2) [39], and later by palladium on activated carbon (Pd/C). These metal catalysts have proven indispensable due to their robustness, scalability, and broad substrate scope and are routinely employed for the reduction of alkenes regardless of their activation status. The limited chemoselectivity can be adjusted by reaction conditions or additives (e.g., pressure, Lindlar's catalyst [40]), and is still topic of active research. For example, a 2025 study showed that tetranuclear Pd clusters on activated carbon deliver markedly improved alkene‐selectivity [41].

**SCHEME 3:**
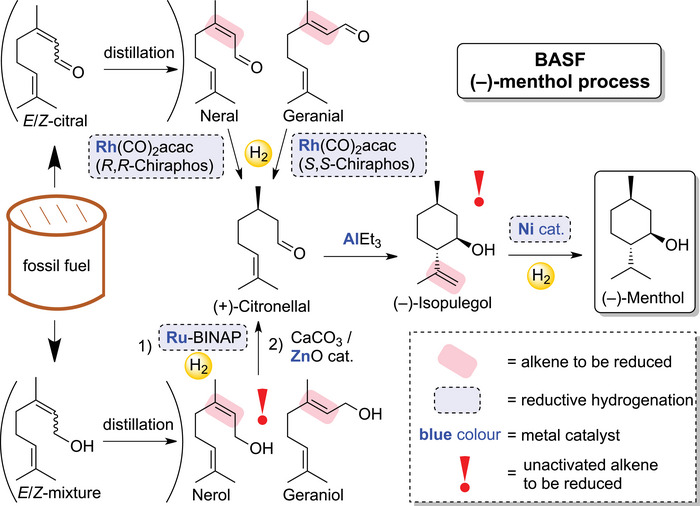
The industrial process of BASF for the production of (–)‐menthol [45, 46].

The transition to molecular defined systems was realized with Wilkinson's homogenous RhCl(PPh_3_)_3_ catalyst [42], which enabled controlled hydrogenation of alkenes under mild conditions (1973 Nobel Prize in Chemistry). Building upon this groundwork, Knowles’ Rh(I)‐based catalysts (1968) [43], and Noyori's Ru(II)‐based systems (1987) [44] introduced asymmetric induction into homogeneous systems, delivering more universally applicable protocols for asymmetric hydrogenation (2001 Nobel Prize in Chemistry to Knowles and Noyori). A prominent industrial implementation by BASF employs these catalysts for the large‐scale production of the flavor compound (–)‐menthol, with annual output reaching thousands of metric tons production scale (Scheme 3) [45]. Remarkably, this process includes two hydrogenation steps involving unactivated alkenes.

Among these developments, Schwartz introduced a hydrozirconation reagent, enabling the transformation of unactivated olefins into organozirconium intermediates that could be subsequently converted into alkanes upon reaction with electrophiles (Scheme 2) [47]. The Buchwald group expanded on this chemistry, demonstrating that titanocene [48] and zirconocene [49] could act as catalysts for reducing tri‐ and tetrasubstituted olefins, although the reported examples were limited to substrates exhibiting benzylic activation. These complexes did not gain widespread use, primarily due to high catalyst loadings.

Crabtree's catalyst represented a major breakthrough for the reduction of sterically‐hindered and unactivated alkenes (Scheme 2) [2]. However, enantioselective variants remained elusive. The Pfaltz group achieved substantial progress by incorporating a bulky counterion and a chiral ligand, which enhanced asymmetric induction while suppressing catalyst degradation [50]. This extended the iridium‐catalyzed asymmetric hydrogenation to trialkyl‐substituted olefins completely devoid of functional groups (Scheme 2) [51]. The Burgess and Andersson groups further refined iridium‐catalyzed hydrogenation, underscoring advances in the reduction of sterically congested alkenes [5, 52]. Although renewable feedstocks can substitute fossil‐derived materials, the reliance on scarce transition metals poses both economic and ecological concerns. Their extraction and refinement are costly and environmentally detrimental, while societal acceptance is declining [53]. Additionally, removing these catalysts is essential in pharmaceutical and food production to ensure safety.

In 2006, frustrated Lewis pairs (FLPs) were shown to cleave H_2_ without metals, extending main‐group chemistry into transition‐metal territory [54]. Despite this conceptual elegance, FLPs are generally limited in scope, robustness, and kinetics, rendering it insufficient for isolated olefins [55]. Concurrently, Chirik group enabled homogenous hydrogenation of unactivated alkenes with low earth‐abundant metal catalyst (e.g., Fe, Co) loadings [56, 57]. The reliance on H_2_ gas remains a limitation.

**SCHEME 4:**
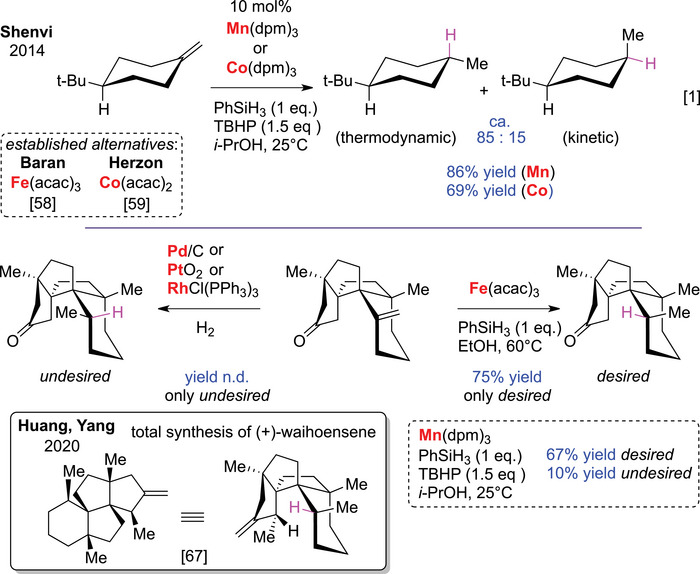
Addition of hydrogen atom transfer (HAT)‐based methods to the chemical toolbox for reducing unactivated alkenes.

Shenvi and coworkers advanced hydrogen‐free reductions using Mn(dpm)_3_ or Co(dpm)_3_ catalysts with phenylsilane as the hydrogen donor (Scheme 4) [1]. Operating via an HAT mechanism, this approach enables mild reduction of unactivated alkenes through tertiary carbon‐centered radicals, which are energetically more accessible than radical anions (Figure 2).

Building on prior reports [58, 59], the Shenvi group harnessed the mechanistic power of HAT to address a long‐standing challenge controlling selectivity in unactivated alkene reductions, where steric effects favor kinetic over thermodynamic products. Conventional hydrogenations with Pd/C or Crabtree's catalyst typically afford poor stereoselectivity for substrates like 1,4‐disubstituted cyclohexenes. Shenvi's system accesses the thermodynamically preferred isomer via radical intermediates, with the added benefits of high abundance and low toxicity of Mn and Co. Concurrently, the Baran [60] and Herzon [61] groups expanded Fe‐, Co‐, and Mn‐based HAT catalysis, originally evolved by Mukaiyama [62], Boger [63], and Carreira [64], using Fe(acac)_2_ and Co(acac)_2_ for related hydrofunctionalization reactions, which also proved effective for unactivated alkene reduction. Collectively, these advances revitalized radical‐based hydrogenation chemistry and established earth‐abundant first‐row metals as practical catalysts for chemoselective alkene reductions [7, 9, 10, 65]. The ability of HAT‐based methods to favor thermodynamic products has been particularly valuable in complex natural product syntheses [9, 66]. Qu et al. observed in the total synthesis of (+)‐waihoensene (2020) that traditional hydrogenations yielded the undesired kinetic isomer due to convex‐face reduction of a sterically encumbered alkene (Scheme 4) [67]. HAT conditions delivered the desired diastereomer as the major or exclusive product.

The Harder group broadened unactivated alkenes reduction by introducing alkaline earth metals. Barium amides (Ba[N(SiMe_3_)_2_]_2_) activate H_2_ to achieve high conversions under elevated temperatures and pressure [68, 69]. Finely divided Fe^0^ synergistically enhanced barium's hydrogenation activity [70]. In fact, stoichiometric LiAlH_4_ with Fe^0^ also enabled reductions [71]. While mechanistically insightful, the harsh conditions and stoichiometric reagents limit practical use.

## 4 Enzymatic Reduction of Unactivated Alkenes

### 4.1 Nature's Strategies

In recent decades, biocatalysis has emerged as a versatile and sustainable synthetic strategy, driven by directed evolution [72] pioneered by Stemmer, Arnold, and others (2018 Nobel Prize in Chemistry) [73]. Many traditional chemical processes, including industrial‐scale reactions [19, 74], have been replaced by biocatalytic transformations, establishing enzymes as complementary and synergistic tools in synthesis [17, 18]. Despite this progress, enzymatic alkene reductions remain largely confined to activated alkenes with EWGs, such as those processed by ene‐reductases (EREDs), even though nature regularly manipulates unactivated alkenes. This gap is reflected in the literature: only two reviews specifically discuss enzymatic unactivated alkene reduction [3, 75], versus many devoted to reductases in general.

**SCHEME 5:**
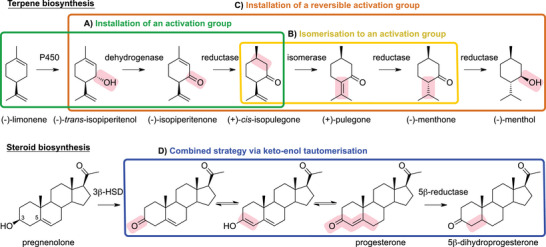
Various strategies for the reduction of unactivated alkenes found in nature: (A) installation of a functional group in conjugation to the alkene to allow reduction; (B) isomerization of the double bond for conjugation to an activation group; (C) reversible installation of an activation group; and (D) combined strategy of A and B by installing an activation group and conjugating it to the alkene via keto–enol tautomerization. Enzymatic transformations are marked with red background.

Natural metabolism offers inspiration, as dimethylallyl pyrophosphate (DMAPP), the universal terpene building block, contains an unactivated C═C bond that remains intact during coupling to form monoterpenes like limonene. In the biosynthesis of (–)‐menthol from (–)‐limonene, no direct reduction of unactivated alkenes occurs. Instead, nature employs indirect activation strategies (Scheme 5) to facilitate reduction. Although only three transformations (two reductions and one hydroxylation) would suffice chemically, the pathway spans six enzymatic steps, suggesting that direct alkene reduction is evolutionarily disfavored. These detours enable greater structural diversification, generating natural products that may enhance organismal fitness or suppress competitors [76]. Careful interpretation is needed for studies using crude lysate, as background reactions such as isomerization or allylic oxidation can confound results [77].

**FIGURE 3:**
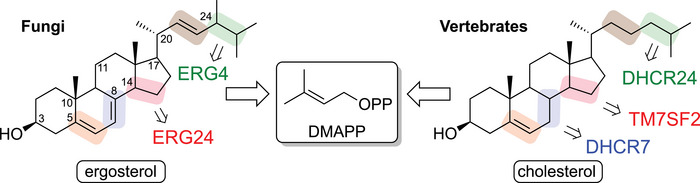
Activities of various reductases toward unactivated alkene functionality in the biosynthesis of steroids.

Direct enzymatic reduction of unactivated alkenes is rare and usually catalyzed by highly specialized reductases within defined biosynthetic pathways. Steroid biosynthesis provides notable examples (Figure 3) [78, 79].

Steroids in fungi, plants, and vertebrates derive from 4,4‐dimethylcholesta‐8,14,24‐trienol, containing three double bonds (Δ8, Δ14, Δ24), of which Δ7 and Δ14 are conjugated. While vertebrates and plants reduce all three, fungi reduce only Δ14 and Δ24. Additional alkenes are introduced and isomerized en route to ergosterol or cholesterol. Precise reduction is essential: deficiencies or malfunctions in these reductases cause severe disorders, such as Smith–Lemli–Opitz Syndrome (Δ7), HEM dysplasia (Δ14), and desmosterolosis (Δ24), highlighting the critical role of these transformations in metabolism [80].

**SCHEME 6:**
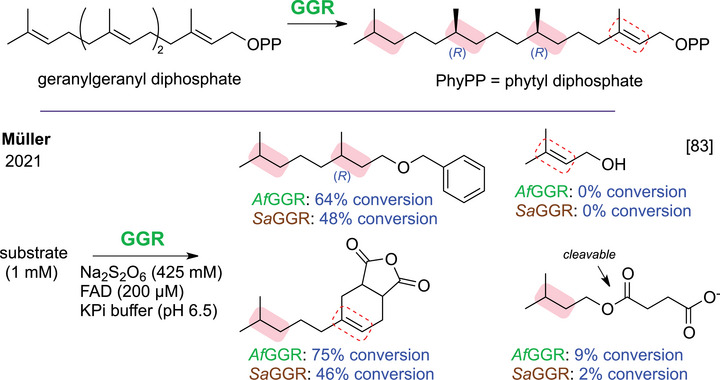
Activity of GGRs on the natural substrate (top) and on structural derivatives (bottom). *Af*GGR = GGR from *Archaeoglobus fulgidus; Sa*GGR = GGR from *Sulfolobus acidocaldarius*.

**FIGURE 4:**
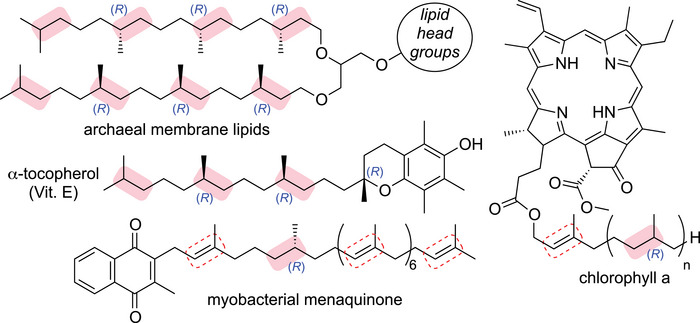
Natural products biosynthesized by geranylgeranyl reductases (GGRs).

Geranylgeranyl reductases (GGRs) reduce multiple C═C bonds in geranylgeranyl chains (*R*)‐stereoselectively while preserving the double bond allylic to the head group. Upon geranylgeranyl pyrophosphate reduction, the phytyl pyrophosphate product subsequently serves as an isoprenoid donor in biosynthetic pathways mediated by prenyl transferases (Scheme 6). GGRs can also act post‐transfer, reducing alkenes after prenylation, though the exact timing within the biosynthetic sequence remains unclear. Many well‐studied GGRs derive from archaeal membrane lipid biosynthesis, notably *Sulfolobus acidocaldarius* (*Sa*GGR) [81, 82], where they fully hydrogenate geranylgeranyl chains to saturated archaeol‐lipids. Müller and coworkers systematically explored derivatives with modified head group and chain lengths, observing partial or full reduction with strict (*R*)‐enantioselectivity (Scheme 6) [83]. They also introduced an in vitro cofactor regeneration system for these enzymes [84]. GGRs also contribute to the biosynthesis of essential metabolites such as α–tocopherol (vitamin E) and chlorophyll a, which feature partially or fully reduced prenyl side chains (Figure 4). In menaquinone (vitamin K_2_) biosynthesis, only the β‐position alkene among nine isolated C═C bonds is reduced [85], prompting studies of MenJ from *Mycobacterium tuberculosis*, which shows broader substrate tolerance in vitro than in vivo [86, 87].

How do these enzymes reduce isolated and electronically neutral alkenes? Direct enzymatic hydrogenation via the classical hydride transfer of reductases can be considered challenging. The nonpolar character and low reactivity of unactivated C═C bonds make typical active‐site residues insufficiently acidic to promote alkene protonation. High‐resolution crystal structures GGRs with bound flavin adenine dinucleotide (FAD) cofactor and lipid substrates revealed the structural basis of this activity [81, 82, 88]. The substrate's polar head group anchors in an anion pocket, while a conserved PxxYxWxFP motif positions the C═C bond within 3.1 Å of the flavin. The alkene's methyl substituent wedged between phenylalanine and tryptophan residues precisely orients the alkene for reduction. This arrangement favors a *syn*‐addition in which both hydrogens originate from FADH_2_. It remains mechanistically unresolved, whether this occurs via a protonation‐first, hydride‐transfer mechanism, or a concerted two‐electron transfer. While structurally analogous to protonation‐initiated reactions in terpene cyclases, no additional activating factors are employed, indicating a distinct mechanism for GGRs [89]. Substrate dissociation after each reduction allows regioselective partial hydrogenation; if bound continuously, all alkenes would be reduced sequentially [81]. Further mechanistic and structural studies are needed to clarify the basis of GGR activity and selectivity.

### 4.2 Toward Synthetically Useful Biocatalytic Reduction of Isolated Alkenes

**SCHEME 7:**
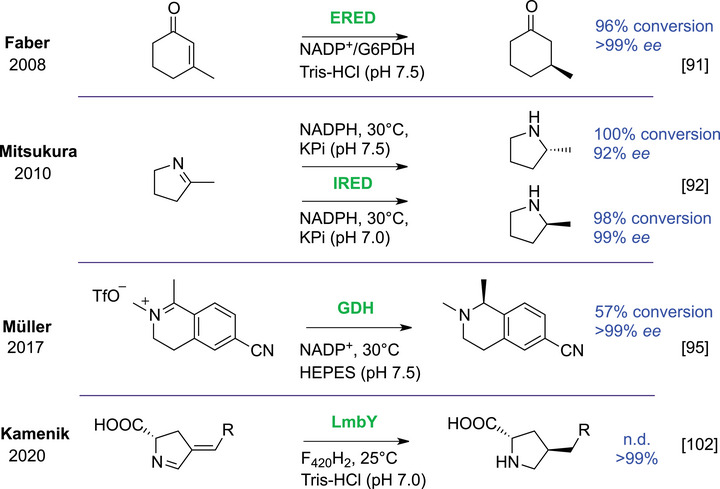
EREDs reduce alkenes activated by an electron‐withdrawing group (top). The discovery of IREDs (second) initiated the search for promiscuous reductases such as the commonly used GDH (third), and the multifunctional reductase LmbY (fourth). G6PDH = glucose‐6‐phosphate dehydrogenase.

Biocatalysis has a long history in reducing activated alkenes and the current zeitgeist seeks enzymatic alternatives to traditional chemical methods. Classical EREDs (formerly “enoate reductase”) [90] have been investigated by Helmut Simon, Faber, and others, before several studies have expanded their scope (Scheme 7) [91]. The discovery of imine reductases (IREDs) in 2010 by extensive screening of 688 microorganisms enabled efficient, asymmetric imine reduction [92] and reductive amination [93], transforming previously limited NAD(P)H‐dependent processes (Scheme 7) [94]. This triggered a cascade of discoveries through serendipitous findings [95], re‐examination of enzymes beyond their known scopes [96, 97], and identification of novel enzyme families [98].

Simultaneously, a notable shift has occurred in the understanding of reductases; historically defined by the concept of specificity, these enzymes are now recognized for their catalytic versatility and promiscuity [75]. The commonly used glucose dehydrogenase (GDH) was observed to possess promiscuous reductive activity (Scheme 7) [95, 99], inspiring its engineering for gram‐scale reductive amination [100]. It is an indication that also other enzymes may have a natural reducing ability and encompass more than they are classified as. Multifunctional reductases converting distinct electrophiles have also been revealed [101, 102, 103]. For example, underutilized cofactor F_420_H_2_ [102, 104] possesses a stronger reducing potential than NAD(P)H and FADH_2_ [105, 106] and was found in the reductase catalyzing an unusual conjugate reduction (Scheme 7) [102].

**SCHEME 8:**
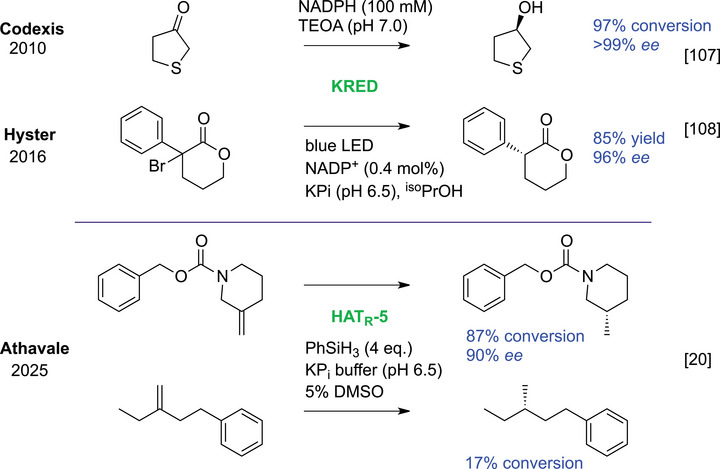
The natural activity of KREDs (top) can be deviated with photo‐induction to novel activity (second). By engineering a hemoprotein, the reduction of unactivated alkenes using an HAT‐mediated mechanism was enabled in an enzymatic environment.

Reductases such as ketoreductases (KREDs) (Scheme 8) [107] can be reprogrammed for transformations beyond classical hydride transfer. In a radically new approach, the Hyster group enhanced cofactor reactivity via photoexcitation, unlocking new‐to‐nature activity in KREDs based on radical‐driven single‐electron transfer (Scheme 8) [108]. ERED‐photocatalyst combinations enabled enantioselective deacetoxylation [109] and keto‐reduction [110]. Complementarily strategies have created unnatural reductases by embedding non‐native mechanisms into proteins lacking inherent reducing activity, including myoglobin and anhydrases [111]. By anchoring phenylsilane‐charged metal hydrides (zinc or iron) in protein scaffolds, artificial metalloenzymes enabled the reduction of ketones, imines and electron‐poor alkenes [112, 113, 114].

Despite these advancements, unactivated alkenes could not be targeted by synthetically useful biocatalysts until recently. Inspired by small‐molecule HAT chemistry as shown in Scheme 4, researchers have begun to engineer enzymes to support radical‐based reactivity. In a notable example, cooperative biocatalytic metal HAT (BioHAT) was achieved in heme proteins by introducing a strategically positioned cysteine residue to mediate enantiodetermining H‐atom abstraction (Scheme 8) [20], analogous to the thiol‐mediated HAT‐process reported by West et al. [115]. Directed evolution delivered high enantioselectivity, good conversion, and even gram‐scale whole‐cell reactions. Consistent with the hypothesis of directed evolution that “*you get what you screen for*,” the substrate scope remained limited. Nevertheless, the work highlights a promising entry point toward general enzymatic hydrogenation of unactivated alkenes.

## 5 Summary and Outlook

The reduction of unactivated alkenes has evolved from an early triumph of transition‐metal‐catalyzed hydrogenation to a defining modern challenge. From Adams’ seminal catalysts to Noyori's asymmetric systems, and more recently to electrochemical and radical paradigms, each generation has pursued selective, economical transformations of the most inert π‐bonds. Clear trends toward greater sustainability and operational simplicity have emerged, driven by increased reliance on earth‐abundant metals [116]. Yet, no universal solution has arisen after a century of progress; instead, chemists have assembled diverse mechanistic strategies, each suited to specific substrates and selectivity demands. Collectively, they form a toolbox rather than a hierarchy, and the synergistic integration, particularly of enzymatic approaches.

Radical‐based HAT systems pioneered by Shenvi [1], Baran [60], and others exemplify this diversification, enabling mild and selective functionalization of unactivated C═C bonds. Parallel biocatalytic innovation such as BioHAT begin to transpose these radical motifs into enzymatic contexts [20], while nature's rich repertoire of reductases remains a largely untapped source of new reactivity. Rigid enzyme classifications can obscure this potential: negative results within narrow families should not discourage broader exploration. Nature frequently accomplishes difficult reductions through cofactor cooperation [117] and electron bifurcation [118], offering inspiration for future strategies.

As metal catalysis, radical chemistry, and biocatalysis grow increasingly compatible [7], their convergence is poised to accelerate discovery. Continued progress will rely on uncovering new enzymes, new reactivities, and the full synthetic potential of this long‐standing yet still underexplored transformation.

## Conflicts of Interest

The authors declare no conflicts of interest.
